# Prevalence of HIV and associated risk factors among street-connected children in Mwanza city

**DOI:** 10.1371/journal.pone.0271042

**Published:** 2022-11-08

**Authors:** Said Nyumayo, Eveline Konje, Benson Kidenya, Anthony Kapesa, Marko Hingi, Nyagwegwe Wango, Joshua Ngimbwa, Violeth Alphonce, Namanya Basinda

**Affiliations:** 1 Department of Community Medicine, School of Public Health, Catholic University of Health and Allied Sciences, Mwanza, Tanzania; 2 Department of Epidemiology and Biostatistics, School of Public Health, Catholic University of Health and Allied Sciences, Mwanza, Tanzania; 3 Department of Biochemistry, Weill-Bugando School of Medicine, Catholic University of Health and Allied Sciences, Mwanza, Tanzania; UNITED KINGDOM

## Abstract

While on the street, HIV infection among street-connected children is a challenging issue due to the nature of transmission, distribution, and prevention. Lack of proper care and protection, insufficient knowledge of the danger of acquiring HIV, and insufficient or absence of health facilities serving street-connected children have left this vulnerable group engaging in high-risk behaviors exposing them to acquiring HIV. This cross-sectional study aimed at estimating the prevalence of HIV infection and its associated risk factors among street-connected children aged between 10 to17 years in Mwanza City. The study was granted ethical clearance all permissions and restrictions to work with street-connected children were adhered to. A total of 131 participants aged 10–17 years were recruited for interviews and HIV testing. Exact logistic regression was used to determine factors associated with having HIV. A total of 111 (85.0%) boys and 20 (15.0%) girls responded to the questionnaire, with the median age being 15years. The overall HIV prevalence was 12.2% (16/131). Street-children using condoms were less likely to be affected by HIV compared to those who were not using (OR = 0.24; 95% CI 0.04–0.97). Females had higher odds of HIV infection compared to males (OR = 5.24; 95% CI of 1.24–24.65). The study shows a significantly higher prevalence of HIV among street-connected children as compared to the general population. Therefore, there should be tailored reproductive and sexual health programs, with the provision of protective materials like condoms.

## Introduction

United Nations (UN) estimates that about 150 million street-connected children worldwide are increasing daily [1]. It is a tremendous increase because, in 2004, the number of street-connected children was estimated to be 100 million worldwide [2]. Concerns have been drawn by United Nations International Children’s Emergency Fund (UNICEF) regarding the growing numbers on urban streets in low- and middle-income countries around the world. Street-connected children are often children under the age of 18 years living under challenging circumstances on the streets, as has been identified by the UNICEF [3]. Globally, the rapid increase in the number of street-connected children is said to be associated with poverty, rural-urban migration, rapid urbanization, family disintegration, divorce, natural disasters, civil wars, rapid industrialization, population growth, the HIV/AIDS epidemic, and other challenges of parents/guardians [1, 4]. One of the most shocking factors leading to street-connected children is the high cases of HIV/AIDs among families and communities that lead to parents’ death resulting in the orphanhood. As children lack proper care and safeguard at home, some opt to look for substitute life on the streets, where they also become victims of HIV/AIDS [5]. Apart from that, these children are at threat of many high-risk behaviors such as early initiation of sex, concurrent sex partners, having unprotected sex, abusing drugs by sharing the needles, group sex, sex work, illness due to sexually transmitted diseases, and other infectious diseases, suicide, and physical and mental health problems are the results of living in streets [1, 4, 6, 7]. In addition, high-risk behaviors among street children have severe consequences for the health system and society in the future [8]. Life on the street gener exposes children to HIV/AIDS, and STIs [9]. HIV/AIDS in street-connected children seems to be big problem, especially in developing countries. A study done in Kathmandu City in Nepal reveals that street children were about 5,000 and prevalence of HIV reached 7.6% and factors associated with intravenous drug use and commercial sex. The prevalence is higher compared to the general population which is 0.39% [10]. In Ethiopia, researchers saw that number of street-connected children was estimated to be 500,000–700,000, and the prevalence of HIV was 6.7% compared to the country’s prevalence of 4.7% and 0.6% for urban and rural, respectively. Factors associated with high prevalence were staying on the street for a long time, having no place to sleep, having concurrent sexual partners and inconsistency in condom use [1, 11]. Kenya is said to have about 300,000 street Children, and the prevalence of HIV to this group was estimated to be 6%, while the general population is 6.2%. Glue sniffing and fuel inhalation, unprotected sex, drug use, and lack of information on HIV/AIDS are the factors leading to the problem [4, 5]. In the Tanzanian context, the cities of Dar es Salaam, Arusha, Tanga, Mwanza, Morogoro, and Mbeya are much experienced with the problem of street-connected children [12]. Their number is increasing daily due to poverty, family disintegration, urbanization, HIV/AIDS epidemic, and industrialization [1, 4]. While on the street, these children lack education, care, and protection, health services, awareness programs hence increasing their vulnerability and engaging in risk behaviors for acquiring HIV/AIDS [5, 13]. Streetlife is full of problems and hardship, nowhere to get money, food, and sleeping areas so children involved in transactional sex, heterosexual, homosexuality, alcohol abuse, and drug abuse to earn living but in turn, these behaviors increase their chance of being infected with HIV [14]. Studies in different countries in Sub-Saharan Africa (SSA) revealed that the prevalence of HIV infection in street-connected children ranges from 4.2% to 7.6% [1, 4, 5]. To Tanzania, Demographic Health Survey (TDHS) of 2014, the prevalence of HIV among adolescents (10–19 years) is estimated to be 6% [15]. In Mwanza urban studies have shown that the prevalence of HIV infection among the over 18 years old people is 8.6% [14, 16]. There is limited information regarding prevalence of HIV infection among street connected children in Mwanza City and other regions in Tanzania. Therefore, this limited information makes it challenging to plan and allocate of resources appropriately and devise proper interventions.

## Materials and methods

### Study area and design

This study was a cross-sectional study conducted in Mwanza city. According to the 2012 National population and Housing Census, Mwanza City had a population of 706,453, whereby Nyamagana had 363,452; 177, 812 being males and 185, 640 female, and Ilemela had 343, 001; 164, 718 being males and 178, 283 females [17, 18].

The study collected information on background characteristics, socio-economic status, HIV transmission knowledge, risk behaviors, and HIV status, among 131 street-connected children aged between 7 to 17 years in Mwanza City, Northwestern. The study determined the prevalence of HIV and risk factors associated with HIV infection in all respective age groups. Quantitative methods were used to determine the relationship between HIV infection and its risk factors. Street-connected children were from the Regional Library, Hiteshi Garage, Manoji, and Rock Beach in Mwanza City, whereby they gather for different occasions like food, training, and swimming. The descriptions of the variables were used during the analysis.

### Data collection and sampling

Data on socio-demographic characteristics (e.g., age, sex of the child, place of residents, religion, Tribe, level of education) was collected during a structured interviews schedule. The prevalence of HIV was estimated after the HIV test using Bioline and Unigold as directed by National Algorithm set up by WHO and (MoHCDEC) HIV testing guideline [19–21]. Training of data collectors was conducted, followed by a pilot study which was done at Mbugani Primary School, one of the classes where Railway Children Africa’s Staff used to talk and have different games and sports with street-connected children. Twenty-five children were used to pre-test interview questions, whereby 20 were boys and 5 were girls. Those who were willing to participate in the study were randomly selected and interviewed whereby demographic characteristics and risk factors for HIV acquisition were collected. Data on HIV prevalence and the associated factors was collected through counseling and testing of those who responded. Confidentiality was observed throughout the exercise by excluding names and using the initials of the site concerned and numbers in the questionnaire and bio-line respectively. Tents and some offices were used for testing and counseling.

The study included all street-connected children who were present during data collection. Children who were unable to comprehend the study either by being too young or intoxicated were not included in the study.

Convenient areas to recruit Street connected children were selected. These are areas where most of the street-connected children in Mwanza gather. They include the regional library, Hitesh Garage, Manoji, and Rock Beach found in Mwanza City. Usually 36 to 43 street-connected children visit these sites purposely for wound care (healthcare) and training as well as the availability of food. With the aid of staff from Railway Children Africa and Tanzania Rural Health Movement (TRHM) under the Street Medicine Project and street-connected children’s leaders, participants were assembled. A random selection of 26 children from each gathering site was done to get the required sample size.

### Data analysis

Three sets of results are presented. First, a description of the background variables, socio-economic status, HIV knowledge, risk behaviors, and HIV infection. The second was to examine the factors associated with HIV infection using the exact test. Last, an exact logistic regression model was fitted to study the relationship between an outcome of interest and the independent variables. These measures have been identified as important predictors of HIV risk behaviors. The exact logistic regression was performed because our outcome variable of interest (HIV infection) is binary, the sample size is small, and some cells were empty. The models were analyzed in three steps; the first model included children’s HIV acquisition and condom use, night-spend, alcohol, and drug use; then the second model included all the variables in the first model plus socio-demographic variables (sex, age, education, occupation on the street, income, duration on the street) and HIV knowledge. Other unsafe risk behaviors such as multiple sex partners and homosexuals were included as confounders because multiple sex partners and homosexuals (a man who has sex with men) tend to increase the risks of acquiring HIV infections. Our final model included only the variables of interest and the significant variables from the above three models.

Odds ratios with a 95% confidence interval (95% CI) were computed for the association between factors and HIV infection. All the analyses were done using STATA version 14, and statistical significance was considered at a p-value less than 0.05.

### Ethical approval and consent to participate

The research proposal was submitted to the joint BMC/CUHAS ethical review Committee of the Catholic University of Health and Allied Sciences for clearance and approval before being given an Ethical Clearance form no CREC/258/2017. Permission to conduct the study was obtained from Mwanza City Authority (i.e., RAS, City Director, City Medical Officer (CMO), and District Aids Control Coordinator (DACC)). Written informed consent was sought from the Social Welfare Officer of Mwanza City who is the caretaker of these street-connected children before recruitment. After acquiring formal permissions from authorities responsible for the welfare of the children, all children below 18 were asked to assent to being interviewed and later tested for HIV. Participation in this research was voluntary, no one was forced to be interviewed and tested for HIV. No names of respondents were used in this study or during report writing, numbers were assigned to participants to ensure anonymity and personal information remained confidential. CMO and District Social Welfare Officer (DSWO) were informed of the number of streets-connected children found HIV positive during HIV testing and hence link them for further management of these children.

## Results

### Socio-demographic characteristics of participants

A total of 131 (92.3%) street-connected children were interviewed and tested for HIV. There were 111 (85.0%) males and 20 (15.0%) females, aged between 10 and 17 years, with a median age of 15 years. Half (68/131) of children have been on the streets for more than a year, and about 72% (94/131) of children live entirely on the street.

Based on the socio-economic status, 89.3% (117/131) of street-connected children have not completed primary school. Forty-seven percent of children earn less than 2500/- Tanzanian Shillings daily. Half (66/131) of the interviewed street-connected children are involved in carrying items and collecting empty bottles and scrapers. Collecting empty bottles and scrapers is mainly done from the dump in landfills where every type of waste, most of which are harmfully disposed of. They walk with no shoes and collect the items with bare hands, thus become risky to bacterial contaminations. In the other group, 23.7% (31/131) were involved in begging and pickpocketing. In their daily lives, street-connected children and related gangs interact with different groups of people. There were 14.5% (19/131) of the sampled street-connected children involved in washing cars, and buses and helping food vendors with washing dishes. Furthermore, 11.5% (15/131) were involved in sex for money. See Table 1.

**Table 1:** Sociodemographic characteristics of the participants.

Variable	Frequency	Percent
**Sex**		
Male	111	84.7
Female	20	15.3
**Age**		
<15	63	48.1
≥15	68	51.9
**Duration**		
≤1 year	37	28.2
>1 year	94	71.8
**Street life**		
On the street	94	72
Off the street	37	28
**Night spends**		
Spend both day and night	122	85.5
Spend with family	6	4.6
Spend in rented house	13	9.9
**Education level**		
None/Incomplete primary school	117	89.3
Completed primary & above	14	10.7
**Occupation**		
Carrying items &collecting empty bottles and scrapers	66	50.4
Begging & pick pocketing	31	23.7
Sex for money	15	11.5
Washing cars & helping food vendors with washing dishes	19	14.5
**Income**		
≤2500 Tshs	62	47.3
>2500 Tshs	69	52.7

### Prevalence of HIV and risky sexual behaviors among street-connected children

Among 131 street-connected children, the overall HIV prevalence was 12.2% (16/131). About 69% (90/131) of children had ever had sex in their lives, and 12% (15/131) of children are working as sex workers. Moreover, in terms of HIV knowledge and risk behaviors, 87% (114/131) of children were aware of the transmission and knowledge about HIV and awareness of the risky behaviors. Furthermore, frequencies of alcohol and drug use were 61% (80/131) and 70% (92/131), respectively. About 29.8% (51/131) of children were not using condoms when doing sex and about 14% were practicing homosexuality (See Table 2).

**Table 2:** Factors associated with HIV infection among street-connected children in Mwanza.

Variable	HIV status		Fisher’s exact test (p-value)
	Positive	Negative	
**Sex**			
Male	7	104	<0.0001
Female	9	11	
**Age**			
<15	6	57	0.365
≥15	10	58	
**Duration**			
≤1 year	4	33	0.509
>1 year	12	82	
**Street life**			
On the street	15	79	0.028
Off the street	1	36	
**Education level**			
None/Incomplete primary school	13	104	0.233
Completed primary & above	3	11	
**Occupation**			
Carrying items &collecting empty bottles and scrapers	4	62	<0.0001
Begging & pick pocketing	3	28	
Sex for money	2	17	
Washing cars & helping food vendors with washing dishes	7	8	
**Income**			
≤ 2500	6	56	0.285
> 2500	10	59	
**HIV transmission knowledge**			
Yes	14	100	0.685
No	2	15	
**Sexually active**			
Yes	16	74	0.004
No	0	41	
**Sexual partners**			
1	1	24	0.027
>1	15	50	
**Practice homosexual**			
Yes	3	14	0.508
No	13	101	
**Drug use**			
Yes	7	32	0.155
No	9	83	
**Alcohol use**			
Yes	9	32	0.108
No	7	83	
**Condom use**			
Yes	3	37	0.024
No	13	38	
**Night spends**			
Spend both day & night	10	102	0.03

### Factors associated with HIV infection among street connected children

#### Bivariate analysis

In the bivariate analysis, night spending, sex, occupation, street life, sexual activity, sexual partners and condom use were factors associated with HIV infection, see Table 2.

#### Exact logistic regression

After bivariate analysis, both unadjusted and adjusted logistic regression models were performed and only significant variables under a 0.05 significance level were kept in the final model. In the adjusted logistic regression children who were spending their lives completely on the streets were less likely to be affected by HIV compared to those who were spending their lives in the rented houses (staying together with other street-connected children) (AOR = 0.17; 95% CI 0.23–0.88). Furthermore, the analysis showed a significant association between gender and HIV infection, with female having higher odds of HIV infection than to males (AOR = 5.24; 95% CI 1.24–24.65), see Table 3.

**Table 3:** Exact logistic regression results for HIV infection.

Variables	Unadjusted model		Adjusted model	
	Or [95% CI]	p-value	OR [95% CI]	P-value
**Condom use**				
No	1.00			
Yes	0.24 [0.04–0.97]	0.0449	0.49 [0.07–2.70]	0.5544
**Night spends**				
Rented house	1.00			
Spend with day & night	0.12 [0.03–0.51]	0.0034	0.17 [0.23–0.88]	0.0324
Spend with family	0.18 [0.00–1.55]	0.1265	1.12 [0.00–15.94]	0.9999
**Alcohol use**				
No	1.00			
Yes	0.45 [0.13–1.47]	0.2159	0.83 [0.22–3.08]	0.9848
**Drug use**				
No	1.00			
Yes	0.50 [0.15–1.72]	0.3105	0.83 [0.21–3.32]	0.9966
**Sex**				
Males	1.00			
Females	2.46 [1.17–3.82]	<0.0001	5.24 [1.24–24.65]	0.0208
**Age**				
<15years	1.00			
≥15yrs	1.63 [0.50–5.84]	0.05257	-	-
**Education**				
None/Incomplete Primary	1.00			
Complete primary	2.17 [0.34 = 9.81]	0.4658	-	-
**Practice homosexual**				
No	1.00			
Yes	2.17 [0.34–9.81]	0.6874	-	-
**Sex**				
One	1.00			
More than one	7.1 [0.97–315.5]	0.0543	-	-

## Discussion

### The prevalence of HIV

Being the first of its kind in the Mwanza region, was designed to a) assess the HIV status of a sample of street-connected children of Mwanza city and b) identify risk factors associated with HIV infection among these children. The serological tests revealed an HIV prevalence rate of nearly 12% among the sample of street-connected children surveyed. This is over two times greater than the estimated HIV prevalence of 4.7% for the general population of Tanzania [22]. Our findings may reveal the dramatic increase in the number of street-connected children with the possibility of an increase in the prevalence of HIV/AIDS and its complication. As children lack proper care and safeguard at home, some opt to look for substitute life on the streets where they also become victims of HIV/AIDS [5]. Apart from the findings from this study, globally, there is a rapid increase in the number of street-connected children which is said to be associated with poverty, rural-urban migration, rapid urbanization, family disintegration, divorce, natural disasters, civil wars, rapid industrialization, population growth, the HIV/AIDS epidemic and other challenges of parents/guardians [1, 4].

### Unprotected sex

Condom is one of the contraceptives used to prevent early and unexpected pregnancies and transmission of STIs including HIV. More than half of the interviewed street-connected children claimed that they didn’t use condoms during sexual intercourse. As reported in other studies, condoms were used when doing sex for money by 61.1% of girls and 23.1% of boys and this was only when someone providing money decided to use them [23]. However, Girls also reported that it becomes difficult to *negotiate* with your clients about using *condoms* when their client are unwilling in use them, especially those men from outside the street life. This leads to the street children only using condoms when clients need them [24]. These habits predispose children to HIV/AIDS acquisition. This can be accounted for by the lack of access to free condoms and that they cannot afford to buy condoms. Other authors say that the absence of caregivers leads to children being unprotected, poverty is taken as one of the major risk factors for contracting HIV whereby children fail to get basic needs in life such as food, clothes, education, hence causing them to be abused and exploited [25]. Due to social-economic similarity, there are almost similar higher findings on condom use among street-connected children in Dar es salaam Amaury et al 2010 and our study [9]. The results from the statistical analysis show that children who were using condoms were less likely to be affected by HIV compared to those who were not using them. This observation is similar to the one done by stojadinovic in their study of sexual behavior of street-connected children found the same results that the majority of street-connected children do not use a condom during sexual intercourse, and unprotected sex has a significant effect on HIV prevalence [26]. The act of doing unsafe anal sex, vagina sex, and having more than one sexual partner expose them to HIV infection [9, 27, 28]. Tender age means no power for decision-making. Many street-connected children drop school or fail to join school totally, with a low level of education sometimes they even miss essential knowledge on HIV/AIDS, which in turn makes them fail to make an informed decision concerning their health hence contracting HIV [5, 6, 16].

### Drug use

Drug use is dominant among street-connected children in our current study. Marijuana, drinking alcohol, and sniffing glue which was mostly used by older boys and girls of the age of 14 and above in Mwanza. The younger boys claimed not to use any kind of illicit drug but rather smoke cigarettes. Drug use increases the chance of contracting HIV. Most drug users share injections and are primarily used in groups in which if one individual is HIV positive, the other members can be easily affected.

Furthermore, a study done in Mwanza by *Mary et al 2014* showed that drugs result in the loss of personal control as it affects the brain, lead to the engagement of unprotected sexual intercourse, and anal sex, and increase the cases of rape [29]. Despite the fact that the interviewed street-connected children know well about the association between drug use and the chance of contracting HIV, most of them reported that due to the complex and helpless life they pass through, they do engage in drug use and alcoholism to reduce stress and make them sleep well. As reported in other studies, drug-using increases significantly the risk for HIV&AIDS [1, 4, 6, 7]. Girls further reported that drugs increase their confidence in the business of sexual work during the argument process with the customers. The association between drug use and HIV was checked, but it was not statistically significant.

### Alcohol use

When participants were asked how does alcohol influences HIV prevalence among street-connected children, the majority explain that there is no relationship between taking alcohol and HIV prevalence because; alcohol helps them become relaxed and sleepy, while few argued that alcohol consumption increases the risks of contracting HIV because it weakens the reasoning ability to notice the risky things like sharing of sharp equipments. Moreover, one participant provided an example that, when young street-connected boys get intoxicated, older boys take advantage to practice anal sex (homosexual) with them, hence increasing the risk of contracting HIV. The relationship between alcohol use and HIV infection was checked, but it wasn’t statistically significant [1, 4, 6, 7].

### Gender distribution and existence of female sex workers

Out of the sampled population, only 15.3% were girls while the majority (84.7%) were boys. Similar results of the biased gender distribution among street-connected children are supported by other studies of street-connected children in the cities of Tanzania. Most studies on street-connected children in Tanzania suggest that girls only represent an estimated 20 to 30% of the total number of street-connected children [30–32]. Tanzania and many other African countries experience more boys in the streets as street-connected children than girls is that traditional cultural values restrict girls’ freedom of movement compared to boys. Thus, many girls are in domestic employment, and not working openly on the streets [30]. The exploitation of girls as domestic workers is linked to the smaller proportion of girls living independently in the street environment.

However, a small portion of girls in the streets experience more pain in the street life than boys. Most of them are being used by boys for sexual pleasure. They cannot fight the same way as boys and therefore, they are forced to exchange protection, shelter, and clothing for sex and engage in sex businesses by taxi drivers and other older people.

The study participants reported a high existence of female sex workers among street-connected children, which leads to the high prevalence of HIV. About 15% of street-connected girls reported engaging themselves in the sex business. On the other hand, several male participants reported that female sex workers *(dada poa)* increases the risk of HIV among street-connected boys. It is reported that, sex workers influence street-connected boys through their half-naked dresses, making it difficult for them to abstain. On the other side, girls participants reported that being a sex worker increases the chance of contaminating HIV. This is because they have to engage in sexual intercourse with more than one customer to satisfy their needs, which places them at a high risk of contaminating HIV. The analysis also showed a significant association between gender and HIV infection, with the female having higher odds of HIV infection compared to males.

### Homosexual activities and sex partners

In General, the results indicate that 13% of the respondents are engaged in homosexual practice. Homosexuality is one of the ways that can easily spread STDs and HIV because of the high friction during anal sex. This action needs early stop initiations, once the children are used to it, it becomes difficult to stop as they become addicted. Contrary to Mandalazi and Umar (2017), this study find a much lower HIV prevalence among children who were practicing homosexuality as compared to those who were not [33].

The results further show that most children have more than one sexual partner, and the HIV prevalence was much higher among children with more than one sexual partner. This shows a built-up network of sex relationships among the children themselves and other people their age. Despite the argument by the majority that they have the knowledge of the HIV transmissions but it seems they are not active in taking precautions in sexual relationships to keep themselves safe. The older boys can have more than one little girl [9, 27, 28].

### HIV knowledge

Daily, street-connected children faced constant violence, which goes hand in hand with the risks linked to drug-taking and infection by sexually transmitted diseases (STDs). In addition, they are particularly exposed to the human immunodeficiency virus (HIV) and the acquired immunodeficiency syndrome (AIDS). It is, therefore, important for street-connected children to be imparted with the knowledge of HIV transmission. The results show that the majority (87%) of the street-connected children knew about HIV transmission knowledge indicating that the knowledge is spread among the street-connected children and that majority of them are safe from the transmission. However, in contrast to other studies that have shown a low level of education among street-connected children, sometimes they even miss important knowledge on HIV/AIDS which in turn makes them fail to make an informed decision concerning their health hence contracting HIV [5, 6, 16]. Furthermore, the use of audiovisual devices in disseminating HIV knowledge has made it practical and useful since most street-connected children cannot read and write therefore, the devices such as radios and Televisions are the best options that effectively work.

The children explained that their knowledge about HIV helps them to be aware and careful about HIV risk behaviors. They mentioned that they get information from health facilitators from organizations who visit them in the street. Some of them got their information when they attended primary education. HIV knowledge helps them to avoid practicing anal sex, sharing sharp tools, and having more than one partner. However, there is a need for care in determining the actual knowledge that street-connected children have. They may share preconceived ideas concerning the virus, how it is transmitted, how to treat it, or how to protect against any risk of infection which may or may not be proper.

## Conclusion

The overall HIV prevalence was more than two times higher in Street connected children (12.2%) as compared to the general population (4.7%). A female street-connected child is more vulnerable to contracting HIV/AIDS. All street children are prone to a lack of use of protection during sexual intercourse. Tailored programs for street-connected children-friendly sexual and reproductive health services need to be initiated to cater to the need for protection and overcoming vulnerability.
